# Ecological Speciation in *Nolina parviflora* (Asparagaceae): Lacking Spatial Connectivity along of the Trans-Mexican Volcanic Belt

**DOI:** 10.1371/journal.pone.0098754

**Published:** 2014-06-06

**Authors:** Eduardo Ruiz-Sanchez, Chelsea D. Specht

**Affiliations:** 1 Red de Biodiversidad y Sistemática, Centro Regional de Bajío, Instituto de Ecología AC, Pátzcuaro, Michoacán, Mexico; 2 Departments of Plant and Microbial Biology and Integrative Biology and the University and Jepson Herbaria, University of California, Berkeley, Berkeley, California, United States of America; University of Arkansas, United States of America

## Abstract

The hypothesis of ecological speciation states that as populations diverge in different niches, reproductive isolation evolves as a by-product of adaptation to these different environments. In this context, we used *Nolina parviflora* as a model to test if this species evolved via ecological speciation and to explore current and historical gene flow among its populations. *Nolina parviflora* is a montane species endemic to Mexico with its geographical distribution restricted largely to the Trans-Mexican Volcanic Belt. This mountain range is one of the most complex geological regions in Mexico, having undergone volcanism from the mid-Miocene to the present. Ecologically, the Trans-Mexican Volcanic Belt possesses different types of vegetation, including tropical dry forest; oak, pine, pine-oak, and pine-juniper forests; and xerophytic scrub - all of which maintain populations of *N*. *parviflora*. Using species distribution models, climatic analyses, spatial connectivity and morphological comparisons, we found significant differences in climatic and morphological variables between populations of *N. parviflora* in two distinct Trans-Mexican Volcanic Belt regions (east vs. west). This could mean that the geographically isolated populations diverged from one another via niche divergence, indicating ecological speciation. Spatial connectivity analysis revealed no connectivity between these regions under the present or last glacial maximum climate models, indicating a lack of gene flow between the populations of the two regions. The results imply that these populations may encompass more than a single species.

## Introduction

The ecological speciation hypothesis postulates that incipient species adapt to different niches, which are influenced by the ecological environment (e.g. resources, predators or abiotic factors) [1], ultimately resulting in niche differentiation and leading to speciation. Svensson [1] defines three critical conditions necessary for ecological speciation: 1) niche differences must exist with sufficient magnitude between species such that divergent selection can drive speciation, 2) niche differences must persist for a sufficiently long period of time to allow reproductive isolation to evolve, and 3) ecological differentiation must evolve prior to reproductive isolation such that the ecological differentiation is driving the process. However, even given these criteria, ecological speciation is only one factor in the speciation process, with other geographical processes often contributing to reproductive isolation, and with confounding factors associated with ecological differentiation also having an effect (e.g. allopatric, parapatric, and sympatric modes of speciation; [2], [3]).

The ecological niche concept is most purely applied when the geographical ranges of the species in question overlap in distribution [4], [5]. There are two generalizable modes of speciation that can occur in terms of the niche, each with testable hypotheses in the phylogenetic context: niche conservatism and niche divergence [5]. Niche conservatism is the tendency of species to retain ancestral ecological characteristics, and many aspects of the fundamental niche can be conserved over long evolutionary time scales [6], [7]. As such, closely related species are expected to have more similar niches than would evolve randomly [6]. Niche divergence occurs when new species adapt to new niches as part of the speciation process, such that sister species are more divergent in their niche space than would be expected by chance [5]. In the case of allopatric speciation, niche conservatism could play a role early on as species could retain their ancestral niche preferences in geographic isolation; subsequent ecological divergence between the two species would limit any further gene flow between them [1], [5] and provide mechanisms for continued isolation if their ranges were to overlap eventually. Thus, niche conservatism and ecological divergence can work together, but at different stages in the speciation process [1]. The existence of highly conserved ecological niches between closely related species indicates that many taxa rely on the conservation of ancestral niche space and its effects in causing population fragmentation and allopatry, and that this is an important force in speciation [8], [9]. The niche conservatism hypothesis proposes that species distribution patterns are governed by ancestral climatic affinities [10].

Recent studies have proposed new methodologies to test the importance of niche conservatism and niche divergence in speciation [5], [11], [12], particularly using GIS-based environmental datasets. Using these methods, researchers have found evidence for speciation by niche conservatism in temperate montane regions [9], [12]) and in the tropics [5], [6], [13]), or for niche divergence along a climatic gradient in the tropics [14]–[16].

Variations in climate and landscape have been shown to play an important role in divergence at the population level [17]. Global climatic cycles (e.g. glacial maxima) force populations to retract or spread with decreasing or increasing habitat availability, and different species respond differently to these changes [17]. Species with distributions in temperate habitats and at low elevations tend to retract as they lose habitat during glacial maxima [18], causing restricted gene flow among the remaining populations. Species with populations distributed at higher elevations tend to expand their ranges, and thus gene flow is increased among populations with the expansion of suitable habitat [17], [19], [20]). Thus, landscape changes in response to climate change can result in the formation of patches of distribution scattered among optimal habitats [17]. The resulting gaps in distribution lead to a reduction in gene flow among fragments and permit genetic isolation [21].

**Figure 1:**
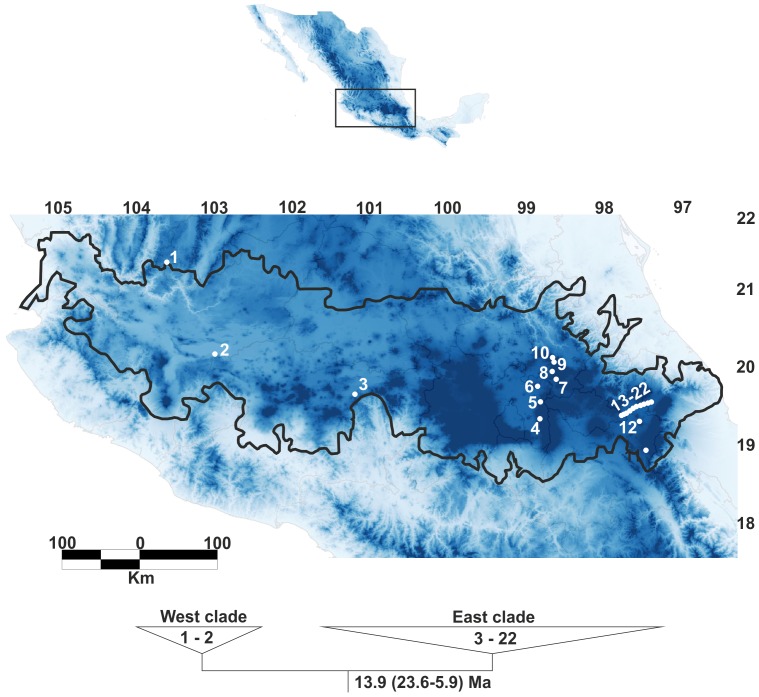
Geographical distribution of *Nolina parviflora* along the Trans-Mexican Volcanic Belt. Population numbers are those given in Table 1. The bottom tree shows the results of the Bayesian molecular dating analysis of *N. parviflora*, numbers inside each clade indicate the populations included in the analysis, and node numbers indicate divergence time and its 95% highest posterior density (HPD) intervals (adapted from Ruiz-Sanchez & Specht, 2013).

*Nolina parviflora* (Kunth) Hemsl., is an excellent model to test if ecological speciation occurred via niche conservatism or niche divergence, and to explore current and historic gene flow among populations. *Nolina parviflora* (Nolinoideae, Asparagaceae) is a montane species endemic to Mexico, with its geographical distribution mainly along the Trans-Mexican Volcanic Belt (TMVB) in central Mexico (Fig. 1), where it inhabits tropical dry, oak, pine, pine-oak, pine-juniper forests and xerophytic scrub from 1700 to 2800 m above sea level [22]. The TMVB is a complex geological region with a high degree of topographic complexity due to its volcanic activity from the mid-Miocene to the present [23], [24]. Recent phylogenetic and phylogeographic studies show that the geological complexity of the TMVB is correlated with the diversification and speciation of various plant and animal lineages [20], [22], [25]–[27].

Our previous phylogeographic study of *N. parviflora* using plastid DNA markers distinguished two well-supported clades separated geographically along the TMVB [22]. Clade 1 comprises populations distributed in the western region of the TMVB, while Clade 2 comprises the remaining populations. Divergence time estimates for these clades indicate a late Oligocene to Pliocene divergence, coinciding with two major volcanic episodes along the TMVB [22]. Our goal in this study is to understand how volcanism influenced ecological speciation in *N. parviflora* and to test whether volcanic events during the Last Glacial Maximum (LGM) modified the landscape and prevented ecological connectivity among population of this species along of the TMVB. Given the current distribution of populations and the ancient divergence between the two major clades, we are particularly interested in investigating the role of niche divergence in incipient speciation and population segregation by investigating the influences of historical climate change, volcanism, and low dispersal ability on current and historical patterns of gene flow in this plant species.

## Materials and Methods

### Genetic data collection

The DNA sequences of two chloroplast spacers [*psbA*-*trnH* (JX178963–JX178986) and *trn*L-F (JX178987–JX179010)], as published previously by Ruiz-Sanchez & Specht [22], were sampled from 22 populations for a total of 158 individuals. Populations extend across the full range of *Nolina parviflora* along the Trans-Mexican Volcanic Belt (Table 1, Fig. 1).

**Table 1:** Geographic location and population code for the 22 *Nolina parviflora* populations studied. Populations located west of the TMVB are indicated by an asterisk (*). TMVB  =  Trans-Mexican Volcanic Belt.

Population	Locality	Code	N Latitude	W Longitude	Elevation(m a.s.l.)	*S*	*h*	π ± SD	Haplotype (*n*)
1	México, Zacatecas, Teul de González*	ZAC	21°21′	103°34′	1708	15	0.755	0.00482±0.00291	H2(5) H3(2) H9(1) H10(1) H11(1)
2	México, Jalisco, Tizapan el Alto*	JAL	20°09′	102°56′	1714	8	0.2	0.00144±0.00108	H2(9) H3(1)
3	México, Michoacán, Morelia	MICH	19°39′	101°08′	2050	2	0.4	0.00082±0.00081	H1(4) H5(1)
4	México, Edo. México, Ixtapaluca	MEX1	19°20′	98°46′	2800	3	0.8	0.00034±0.00048	H5(5) H4(1)
5	México, Edo. México, Tepetlaoxtoc	MEX2	19°33′	98°45′	2660	1	0.285	0±0	H1(7)
6	México, Edo. México, Pirámides	MEX3	19°45′	98°48′	2668	0	0	0±0	H1(7)
7	México, Hidalgo, Tlanalapa	HID4	19°50′	98°33′	2521	0	0	0±0	H1(7)
8	México, Hidalgo, Zempoala	HID3	19°56′	98°36′	2460	1	0.285	0±0	H1(7)
9	México, Hidalgo, Epazoyucán	HID2	20°03′	98°35′	2667	0	0	0±0	H1(7)
10	México, Hidalgo, Epazoyucán	HID1	20°07′	98°36′	2639	0	0	0±0	H1(7)
11	México, Puebla, Ciudad Serdán	PUE9	18°55′	97°24′	2357	3	0.285	0±0	H7(7)
12	México, Puebla, Guadalupe Victoria	PUE10	19°31′	97°25′	2357	1	0.2	0±0	H6(10)
13	México, Tlaxcala, Cuapiaxtla	TLAX	19°17′	97°30′	2384	0	0	0±0	H6(7)
14	México, Puebla, Libres	PUE8	19°23′	97°41′	2465	2	0.523	0±0	H6(7)
15	México, Puebla, Libres	PUE7	19°24′	97°39′	2307	2	0.333	0±0	H6(6)
16	México, Puebla, Libres	PUE6	19°26′	97°36′	2251	0	0	0±0	H6(7)
17	México, Puebla, Tepeyahualco	PUE5	19°28′	97°34′	2261	1	0.285	0±0	H6(7)
18	México, Puebla, Tepeyahualco	PUE4	19°29′	97°31′	2294	13	0.866	0.00239±0.00174	H6(3) H7(2) H8(1)
19	México, Puebla, Tepeyahualco	PUE3	19°30′	97°28′	2337	0	0	0±0	H6(7)
20	México, Puebla, Tepeyahualco	PUE2	19°31′	97°25′	2357	12	0.523	0.00117±0.00097	H6(6) H8(1)
21	México, Veracruz, Perote	VER1	19°32′	97°28′	2334	11	0	0±0	H6(7)
22	México, Veracruz, Perote	VER	19°32′	97°20′	2292	0	0.571	0.00234±0.00165	H6(3) H7(4)

*n* the number of individuals per haplotype in parenthesis; *S* segregating sites; *h* haplotype diversity; π nucleotide diversity and SD. standard deviation.

### Genetic diversity and parsimony network

Plastid markers (*psb*A-*trn*H and *trn*L-F) were combined as they share maternal inheritance and are inherited as a unit [28]. A statistical parsimony haplotype network of the populations of *N. parviflora* from the TMVB was generated with TCS 1.2.1 [29] with a 95% of probability connection limit and treating gaps as missing data. Genetic diversity (*h*), nucleotide diversity (**π**) and the number of segregating sites (*S*) within populations was assessed using Arlequin 3.11 [30] with the default value of 10,000 permutations. DnaSP v5.10.1 [31] was used to interpret pairwise *F*_ST_ values between populations.

### Ecological niche modelling

Species distribution models (SDM; [32]) were constructed in order to predict the presence of suitable habitats for *N. parviflora* during the Last Glacial Maximum (LGM) and to investigate whether or not range expansion and habitat connectivity are observed between west and east populations of the TMVB. Ten environmental variables with a resolution of 1 km^2^ were considered, derived from temperature and precipitation data obtained from WorldClim 1.4 [33]: Mean Diurnal Range, Isothermality, Temperature Seasonality, Max Temperature of Warmest Month, Min Temperature of Coldest Month, Temperature Annual Range, Precipitation Seasonality, Precipitation of Driest Quarter, Precipitation Warmest Quarter and Precipitation of Coldest Quarter. These environmental variables were selected based on their lack of correlation (pairwise r<0.9 based on all sample locations) [34]. Two general atmospheric circulation models (GCM) were used to generate past climate scenarios for the LGM: the Community Climate System Model (CCSM; [35]) and the Model for Interdisciplinary Research on Climate (MIROC; [36]). Both models simulate climatic conditions as they are calculated to have been for the LGM, with a stronger temperature decrease assumed in CCSM compared to MIROC [37]. The original GCM data were downloaded from the PMIP2 website (http://www.pmip2.cnrs-gif.fr/) [38]. The models were run in MaxEnt 3.3.2 [39]; http://www.cs.princeton.edu/~schapire/maxent/). MaxEnt employs a maximum likelihood method that estimates a species' distribution given maximum entropy, subject to the constraint that the environmental variables for the predicted distribution must match the empirical average [39].

A total of 40 georeferenced records, spatially unique and separated from one another by least 1 km, were compiled. Georeferenced data was obtained from our own collections and from the National Biodiversity Information Network (REMIB; http://www.conabio.gob.mx/remib_ingles/doctos/remib_ing.html; accessed Feb 2011). The MaxEnt logistic model output for a given species is a continuous surface of values ranging from 0 to 1, where high values indicate greater habitat/climate suitability for a given species. MaxEnt was run with the default settings for convergence threshold (10^−5^) and 1,000 interactions, ensuring only one locality per grid cell. To evaluate model performance, a random subset of 25% of the total unique records was set aside, and the area under the curve (AUC) of the receiver operating characteristic (ROC) was measured providing a threshold-independent measure of performance. Resulting distributions were projected with QUANTUM GIS 1.8.0-Lisboa. The output of MaxEnt consists of grid maps with each cell having an index of suitability between 0 and 1. Low values indicate that the conditions are unsuitable for the species to occur, whereas high values indicate that the conditions are suitable.

### Climate analysis

From the 40 unique georeferenced records, we conducted two different analyses; (a) multivariate analysis of variance (MANOVA) and (b) the multivariate method introduced in McCormack et al. (2010[12]). A MANOVA was used to test for differences in climate variables among regions of the TMVB, specifically testing for differences between the habitats occupied by eastern vs. western populations. Individual observations were binned into separate groups corresponding to the two clades identified in our previous study [22]. Group 1 ( = Clade 1) contains only populations from the western part of the TMVB, and Group 2 ( = Clade 2) contains all populations located in the eastern part. To examine the overall level of divergence in environmental space among the populations/regions, a Principal Components Analysis (PCA) was conducted using extracted values of the 10 climate variables (WorldClim 1.4; Hijmans et al., 2005[33]) for each unique locality using Quantum Gis v. 1.8.0-Lisboa. A MANOVA of PCA scores (dependent variables) was then performed to test for significant differences among the PCA scores and groups (fixed factors). Post hoc tests (e.g., Tukey's HSD) were used to detect if the regions differed significantly from one another, and if so, for which variables. The PCA and MANOVA analysis were carried out in R [40].

The McCormack et al. [12] method was used to test for niche divergence/conservatism of the eastern vs. western populations of *N. parviflora*. Analyses were performed with QUANTUM GIS 1.8.0-Lisboa. For the McCormack analysis, the occurrence data for two clades were first binned into separate groups, and the values of 10 climate variables were extracted from the occurrence points for each of the lineages. Then, 1,000 random points were generated from within a minimum convex polygon drawn around the occurrence sites, and values of 10 climate variables of the 1,000 random points were extracted as the background predictions from within the geographic ranges of each lineage. A PCA was conducted on these data, extracting the first four PC (niche) axes for further consideration. Niche divergence or conservatism was evaluated on each niche axis by comparing the observed difference between the means for each lineage on that axis to the mean difference in their background environments on the same axis. A null distribution of background divergence was created by recalculating the score of background divergence over 1,000 jackknife replicates with 75% replacement. Significance for rejecting the null was evaluated at the 95% level. All analyses were conducted using Stata 10 [41].

### Spatial connectivity

To generate spatial resistance distances to dispersal among sites based on habitat suitability maps (conductance grid), we imported each SDM (current, CCSM and MIROC) into Circuitscape 3.1 [42]. This program calculates pairwise resistance to gene flow among populations based on all possible paths, not just the least cost path, thus better explaining the movement of genes among widely separated regions over many generations [43], [44]. The input for Circuitscape is a raster data set (habitat map) in which each cell is assigned a conductance value corresponding to the probability of the study organism moving through the habitat type encoded by the cell. We chose a conductance grid in which higher cell values denote greater ease of movement, and applied a connection scheme that allowed gene flow among the four nearest cells. We used the SDM rasters as input maps to quantify pairwise resistance distance between *N. parviflora* populations across its distribution along the TMVB.

To evaluate the influence of climatic (present day, CCSM and MIROC niche models) factors on *N. parviflora* connectivity and genetic structure, pairwise resistance values were generated for our three models (present, CCSM and MIROC) given the pairwise plastid genetic distances (*F*_ST_/(1-*F*_ST_) [45]; Table 2). A Mantel test [46] was conducted to detect any significant associations between plastid pairwise genetic distances and pairwise resistance distances [17]. Mantel correlation coefficients (*r*) were estimated using IBDWS 3.16 [47] with 10,000 permutations.

**Table 2:** Pairwise *F*_ST_ comparisons between pairs of *Nolina parviflora* populations in the TMVB. Populations located west of the TMVB are indicated by an asterisk (*). Site abbreviations are those used in Table 1. Significant values at *P*<0.05 are in bold.

	HID1	HID2	HID3	HID4	JAL*	MEX1	MEX2	MEX3	MICH	VER1	PUE10	PUE2	PUE3	PUE4	PUE6	PUE7	PUE8	PUE9	TLAX	VER	ZAC*	PUE5
HID1	0.00																					
HID2	0.00	0.00																				
HID3	0.00	0.00	0.00																			
HID4	0.00	0.00	0.00	0.00																		
JAL*	**0.91**	**0.91**	**0.91**	**0.91**	0.00																	
MEX1	0.03	0.03	0.03	0.03	**0.90**	0.00																
MEX2	0.00	0.00	0.00	0.00	**0.91**	0.03	0.00															
MEX3	0.00	0.00	0.00	0.00	**0.91**	0.03	0.00	0.00														
MICH	0.07	0.07	0.07	0.07	**0.88**	0.02	0.07	0.07	0.00													
VER1	**1.00**	**1.00**	**1.00**	**1.00**	**0.93**	**0.93**	**1.00**	**1.00**	**0.84**	0.00												
PUE10	**1.00**	**1.00**	**1.00**	**1.00**	**0.94**	**0.94**	**1.00**	**1.00**	**0.87**	0.00	0.00											
PUE2	**0.71**	**0.71**	**0.71**	**0.71**	**0.89**	**0.65**	**0.71**	**0.71**	**0.52**	0.00	0.05	0.00										
PUE3	**1.00**	**1.00**	**1.00**	**1.00**	**0.93**	**0.93**	**1.00**	**1.00**	**0.84**	0.00	0.00	0.00	0.00									
PUE4	**0.49**	**0.49**	**0.49**	**0.49**	**0.84**	**0.43**	**0.49**	**0.49**	**0.30**	0.40	**0.47**	0.09	0.40	0.00								
PUE6	**1.00**	**1.00**	**1.00**	**1.00**	**0.93**	**0.93**	**1.00**	**1.00**	**0.84**	0.00	0.00	0.00	0.00	0.40	0.00							
PUE7	**1.00**	**1.00**	**1.00**	**1.00**	**0.92**	**0.92**	**1.00**	**1.00**	**0.82**	0.00	0.00	−0.02	0.00	0.36	0.00	0.00						
PUE8	**1.00**	**1.00**	**1.00**	**1.00**	**0.93**	**0.93**	**1.00**	**1.00**	**0.84**	0.00	0.00	0.00	0.00	0.40	0.00	0.00	0.00					
PUE9	**1.00**	**1.00**	**1.00**	**1.00**	**0.91**	**0.93**	**1.00**	**1.00**	**0.86**	**1.00**	**1.00**	**0.83**	**1.00**	**0.49**	**1.00**	**1.00**	**1.00**	0.00				
TLAX	**1.00**	**1.00**	**1.00**	**1.00**	**0.93**	**0.93**	**1.00**	**1.00**	**0.84**	0.00	0.00	0.00	0.00	0.40	0.00	0.00	0.00	**1.00**	0.00			
VER	**0.43**	**0.43**	**0.43**	**0.43**	**0.83**	**0.37**	**0.43**	**0.43**	0.27	0.50	**0.57**	0.22	0.50	−0.14	0.50	**0.47**	**0.50**	0.33	0.50	0.00		
ZAC*	**0.65**	**0.65**	**0.65**	**0.65**	0.10	**0.62**	**0.65**	**0.65**	**0.59**	**0.72**	**0.76**	**0.66**	**0.72**	**0.58**	**0.72**	**0.70**	**0.72**	**0.66**	**0.72**	**0.58**	0.00	
PUE5	**1.00**	**1.00**	**1.00**	**1.00**	**0.93**	**0.93**	**1.00**	**1.00**	**0.84**	0.00	0.00	0.00	0.00	0.40	0.00	0.00	0.00	**1.00**	0.00	**0.50**	**0.72**	0.00

### Morphological comparisons

Morphological comparisons between east and west clades were performed using exemplars from the 22 populations sampled along the TMVB (Table 1, Fig. 1). In addition to specimens collected specifically for this study, herbarium material was examined from IBUG, IEB and XAL. Vegetative and floral characters were measured from living material in the field and from our own collections and historical herbarium specimens. Twelve morphological characters noted to be variable among individuals were scored and compared among populations.

## Results

### Genetic diversity and parsimony network

The total length of the aligned combined plastid markers (*psb*A-*trn*H and *trn*L-F) was 987 bp with 18 polymorphic sites (*S*). Haplotype diversity (*h*) ranged from 0–0.866, reflecting the presence of different haplotypes within each site (Table 1). Nucleotide diversity (π) was low (0.0000–0.00482), indicating little variation between sequences from the same population (Table 1). Indels of 1–5 bp were considered missing data. In general, pairwise comparisons of *F*_ST_ values were high and most significant between the west vs. east/easternmost and east vs. easternmost populations of the TMVB (Table 2).

**Figure 2:**
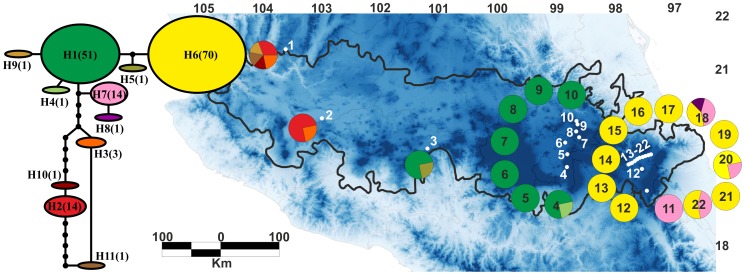
Statistical parsimony network and map of geographical distribution of 11 cpDNA haplotypes found in *Nolina parviflora*. H1-H11: sampled haplotypes. Solid black circles: hypothetical haplotypes. Numbers in brackets indicate individuals. Pie charts represent chloroplast haplotypes for each sampling locality. Population numbers correspond to those in Table 1.

The statistical parsimony network recovered 11 different haplotypes among *N. parviflora* populations. The most frequent haplotype (H6) is shared by populations from Puebla, Tlaxcala and Veracruz, and represents 44.3% of all individuals sampled. This haplotype is geographically distributed in the easternmost part of the TMVB. The second most frequent haplotype (H1) is shared by populations in the states of Edo. México, Hidalgo and Michoacán, and represents 32.2% of all individuals sampled. H1 is geographically distributed in the eastern region of the TMVB. Haplotypes H2 and H3 are shared between the Jalisco and Zacatecas populations in the western region of the TMVB. Haplotypes H4 and H5 are unique to the MICH and MEX1 populations, respectively. Haplotype H7 is shared by the PUE2, 4, 9 and VER populations; and finally haplotypes H8 and H11 are unique to the PUE4 and ZAC populations (Table 1, Fig. 2). Haplotypes H2–3, H9–11 are exclusive to the western part of the TMVB and are not shared with any populations occurring in the eastern area of the TMVB. Haplotypes H1, H4 and H5 are distributed in the eastern region of the TMVB and are not shared with any of the easternmost populations. Finally H6, H7 and H8 are only found in the easternmost part of the TMVB (Fig. 2).

### Ecological niche modelling

**Figure 3:**
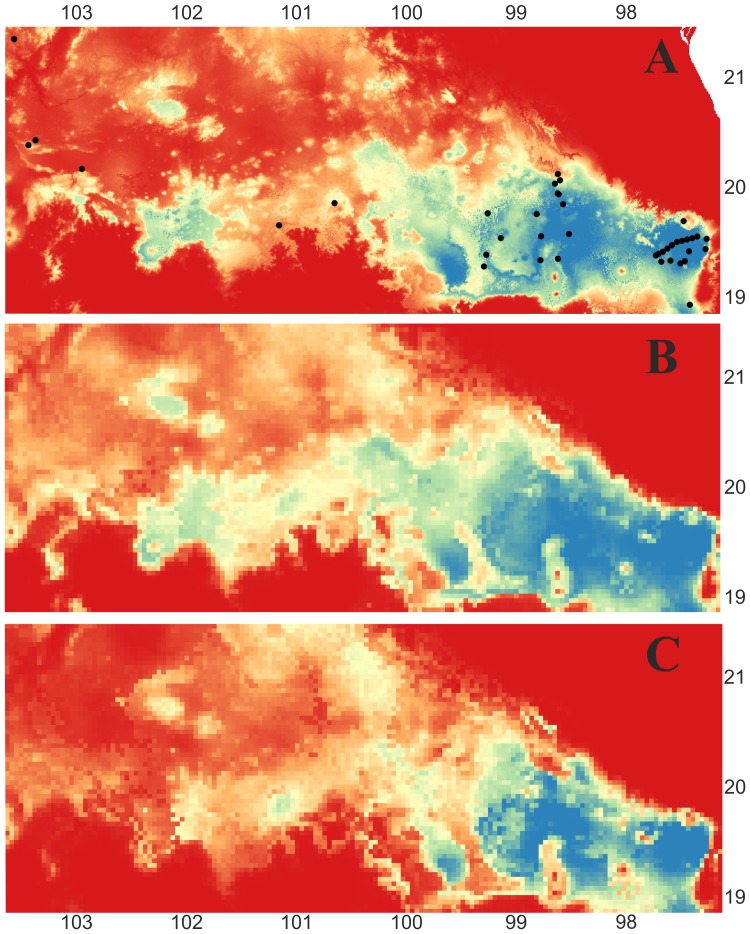
Potential distribution of *Nolina parviflora* identified using species distribution modelling (SDM). A  =  prediction of suitable habitat in the current environment. B  =  prediction projected onto the past climatic layers (LGM; CCSM), and C  =  prediction under past climatic conditions (LGM; MIROC). Blue colour indicates high probability of prediction of suitable habitat and red colour indicates areas with unsuitable habitat.

The modelled distribution (AUC = 0.991) corresponds to the known distribution of *Nolina parviflora* in the TMVB. The SDM for the ten current climate variables across all populations of *N. parviflora* predicted an accurate distribution of *N. parviflora* for the eastern region of the TMVB, but underpredicts the occurrence of populations in the western region of the TMVB (Fig. 3A). When the models were projected onto past climate (21K) layers, two different scenarios were retrieved (Fig. 3B, 3C). For the climate layers based on CCSM (Fig. 3B), a large area of suitable habitat is recovered in the east and easternmost region of the TMVB and extends to the western area of the TMVB beyond the current occurrence. Habitat with some suitability connects these areas. For the projections under MIROC (Fig. 3C) a much smaller area of suitable habitat is predicted and is located mostly in the eastern region, with no connection between the eastern and western predicted areas of suitable habitat (Fig. 3).

### Climate analysis

The PCA of the 10 climate variables found that PC1 = 43.42%; PC2 = 28.63%; PC3 = 17.78% and PC4 = 3.67% together explain 93.5% of the variability. Three temperature and two precipitation variables define PC1 (Table 3); PC2 is defined by Mean Diurnal Range, Isothermality, Min Temperature of Coldest Month, and Precipitation of Driest and Warmest Quarter; PC3 is defined by Mean Diurnal Range, Temperature Seasonality, and Min Temperature of Coldest Month; and PC4 is defined by Max Temperature of Warmest Month and Precipitation of Driest Quarter (Table 3). The PCA/MANOVAs that were performed given two separate regions (West TMVB and East TMVB) detected a statistically significant difference between eastern and western populations (Wilks' lambda  = 0.561; *F*_1-38_ = 6.82; *P* = 0.0003). This difference occurs along PC1 (*F* = 14.34, *P* = 0.0005) and PC2 (*F* = 4.96, *P* = 0.03) indicating that these two regions occur in areas with differences in Mean Diurnal Range, Isothermality, Temperature Seasonality, Max Temperature of Warmest Month, Min Temperature of Coldest Month, Temperature Annual Range, Precipitation Seasonality, Precipitation of Driest, and Warmest and Coldest Quarter.

**Table 3:** Loadings of the environmental variables for each PC axis and tests of niche divergence and conservatism.

	PC1	PC2	PC3	PC4
West vs. East of the TMVB				
BIO2: Mean Diurnal Range	0.237	0.354	0.438	−0.337
BIO3: Isothermality	−0.301	0.371	0.260	−0.265
BIO4: Temperature Seasonality	0.430	−0.202	0.434	−0.240
BIO5: Max Temperature of Warmest Month	0.442	−0.178	−0.338	−0.477
BIO6: Min Temperature of Coldest Month	0.121	−0.675	−0.505	0.202
BIO7: Temperature Annual Range	0.421	0.118	0.276	−0.139
BIO15: Precipitation Seasonality	0.400	−0.170	0.159	−0.106
BIO17: Precipitation of Driest Quarter	−0.181	−0.500	0.186	−0.357
BIO18: Precipitation of Warmest Quarter	0.295	−0.334	0.265	0.174
BIO19: Precipitation of Coldest Quarter	−0.568	−0.266	−0.183	−0.112
Percent variance explained	43.42	28.63	17.78	3.67
Observed difference	3.9701*	**1.7427** *	**0.6412** *	**0.2398** *
Null distribution	4.3129–4.3145	0.8059–0.8087	0. 2900–0.2911	0. 0976–0. 0986

* Significance level, *P*<0.05. Bold values indicate niche divergence.

Observed differences in climatic niche for western and eastern lineages of *Nolina parviflora* on each PC compared to the middle 95th percentile of a null distribution of the differences between their environmental backgrounds.

Tests of niche divergence and conservatism of the four niche axes indicate niche conservatism on niche axis 1(43.42%; Table 3) and niche divergence for axes 2, 3 and 4 (50.08%; Table 3).

### Spatial connectivity

**Figure 4:**
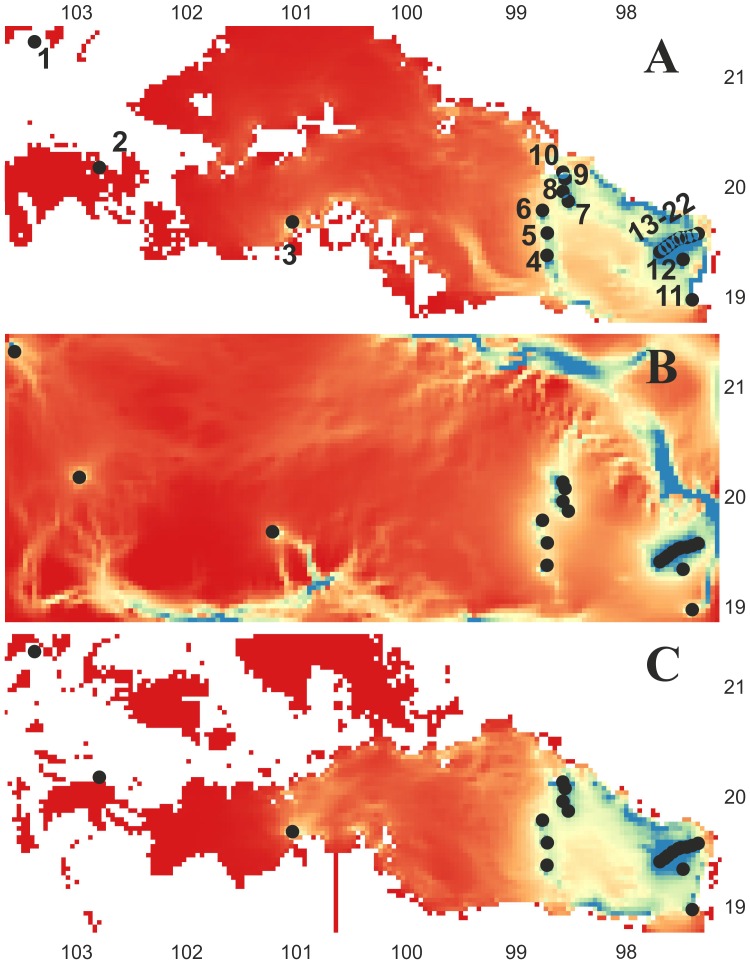
Connectivity maps among populations (black circles) for *Nolina parviflora*. A  =  current environment, B  =  Last Glacial Maximum under the CCSM model, and C  =  Last Glacial Maximum under the MIROC model. Blue and light shades of blue indicate areas with higher current density; areas where connectivity is most tenuous are shown in yellow to red colours.

Climatic spatial resistance surfaces (present, CCSM and MIROC) showed connectivity corridors between neighbouring populations in the easternmost part of the TMVB (populations 4–6; 7–10; 11,12,13–22) for present day conditions (Fig. 4A) and during the last glacial maximum (CCSM; Fig. 4B, MIROC; Fig 4C); but no connectivity was detected between the populations of the eastern and western regions, and likewise no connectivity was maintained between the populations of the western region (Fig. 4) for any of the three climatic spatial resistance surfaces (Fig. 4A,B,C). Spatial genetic correlations showed significant positive correlations between both genetic *F*_ST_/(1-*F*_ST_) pairwise distances and spatial resistance distances among populations in the current or LGM models (present: *r* = 0.27; *P* = 0.005; CCSM: *r* = 0.52; *P* = 0.001 MIROC: *r* = 0.28; *P* = 0.004).

### Morphological comparisons

**Figure 5:**
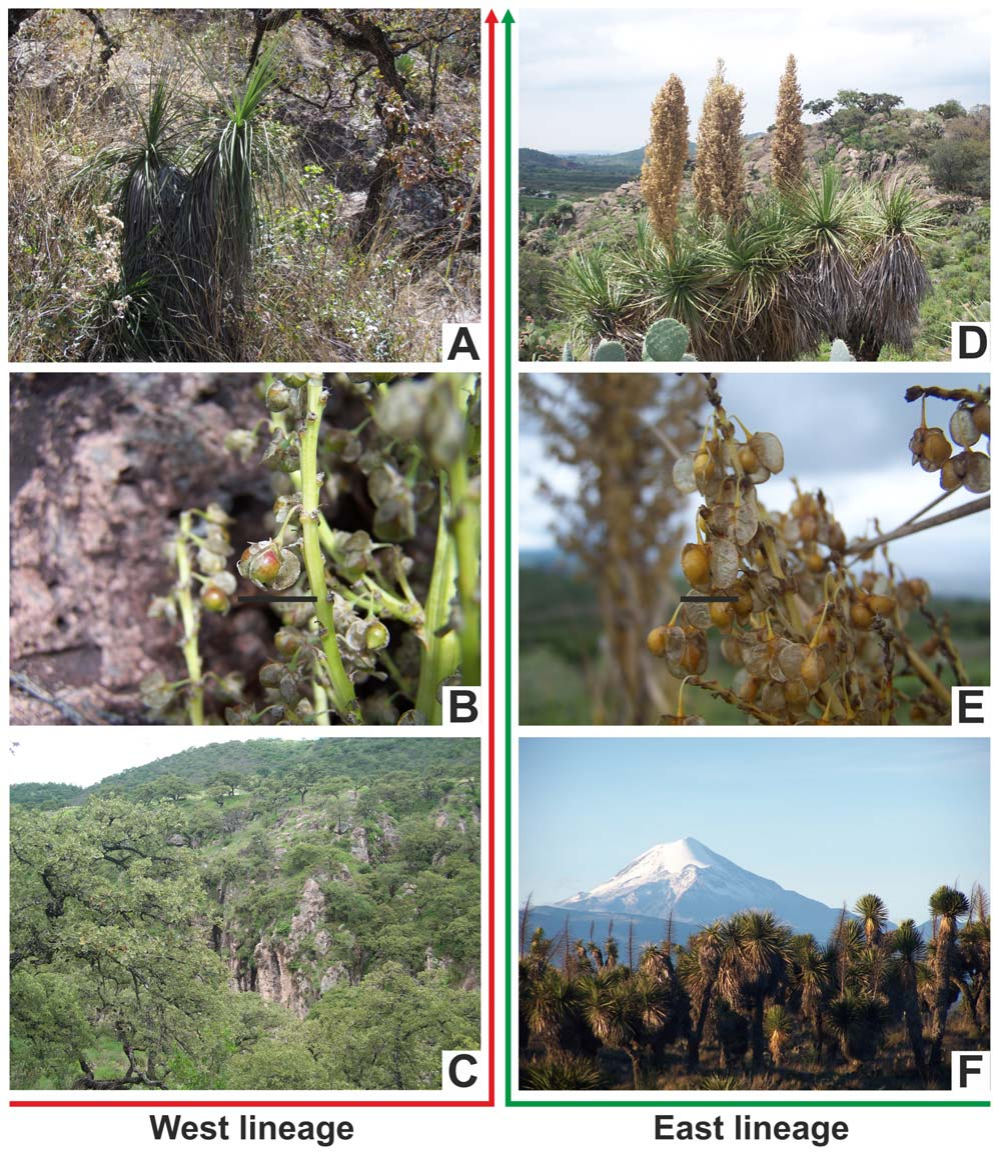
A-C west lineage. A. Plant showing two branches. B. Primary inflorescence branch, showing fruits and seed, scale bar  =  1 cm. C. Panoramic image of the oak forest type in the locality of Río Los Patitos, Zacatecas, Mexico. D-F east lineage. D. Plant showing seven branches and inflorescences. E. Primary inflorescence branch, showing fruits and seeds, scale bar  = 1 cm. F. Panoramic image of xerophytic scrub habitat type with Pico de Orizaba in the background, Veracruz, Mexico. Photos by E. Ruiz-Sanchez.

Twelve vegetative and floral characters noted to vary among individuals within and between populations were scored (Table 4, Fig. 5). When clustered into separate groups, the most significant differences were detected between specimens of the east v. west clades in (1) plant height, (2) leaf length and (3) fruit size (Table 4).

**Table 4:** Vegetative and floral morphological characters scored on specimens from the east and west clades of *N. parviflora* along of the Trans-Mexican Volcanic Belt.

Character	East clade average	West clade average
Total plant height (m)	4–5	1.5–2
Diameter (cm)	10–20	7–9
Number of branches per plant	1 to 6 (8)	1 or 2
Leaf length (cm)	100–150	80–90
Leaf width (mm)	10–20	8–9
Main flowering branch length (cm)	20–30	9–12
Female tepal length (mm)	2.5–4	2.2–2.5
Female tepal width (mm)	1.5–2.5	1.8–2
Fruit length (mm)	7–12	6–6.3
Fruit width (mm)	9–14	7–8
Seed length (mm)	3–4	3.5–4
Seed width (mm)	3–3.5	2–2.5

## Discussion

In this study we integrated analyses of genetic diversity, ecological niche modelling, climate prediction/hindcasting and spatial connectivity to understand how climate, landscape and historical volcanism have influenced ecological divergence and genetic isolation in *N. parviflora* populations distributed along the TMVB. We found a clear east-west pattern of haplotype distribution, where haplotypes H2–3, 9–11 are found exclusively in the western region of the TMVB and the remaining haplotypes are spread throughout the eastern region (Fig. 2). The eastern part of the TMVB is separated by two distinct subregions; the easternmost populations sharing haplotypes H6, 7 and 8 and the eastern populations sharing haplotypes H1, 4 and 5. These two subregions do not share haplotypes between them (Fig. 2). The high values of the *F*_ST_ pairwise comparisons between populations from the east v. west of the TMVB indicate that there is no gene flow between these populations, nor between populations of the two subregions of the eastern TMVB (Table 2). Two recent studies on different species of plants with populations distributed along the TMVB found the same pattern of east-west isolation [48], [49].

### Ecological speciation

Ecological niche models (ENMs) can be used to identify areas of suitable habitat for *N. parviflora* (Fig. 3). ENMs can be projected onto historic climate maps to predict suitable areas for ancient and ancestral distributions, assuming similar habitat preferences were exhibited by historic populations (Fig. 3B, 3C). The ENM for *N. parviflora* given the current distribution underpredicts suitable habitat for some of the known and sampled populations in the western region of the TMVB, even though these populations were used to build the model (Fig. 3A). This could provide evidence for differential habitat preferences among populations, such that segregated populations occupying a different climatic niche could be designated as separately evolving lineages if gene flow is completely lacking between these lineages and those that occupy more central climatic niche space [7]. Another hypothesis is that some ecological factors (e.g. different type of vegetation) may allow for adaptation to different climatic niche spaces, ultimately driving speciation [9], [10]. We observed in the field that *N. parviflora* inhabits very different types of forests, from pine-oak-juniper forests to tropical dry forest, to xerophytic scrub and pine-oak forests. The occupation of different habitats could limit dispersal among populations [50] even if the populations are geographically proximal.

The results of the MANOVAs suggest the occurrence of niche divergence between the populations of the eastern Trans-Mexican Volcanic Belt and those of the western TMVB. In particular, variables associated with temperature and precipitation (PC1 and PC2) differentiate these two regions (Table 3). The populations of the western TMVB are found in tropical dry and oak forests, while those of the eastern TMVB are located primarily in pine-juniper-oak forests and xerophytic scrub. Tests of niche divergence vs. niche conservatism indicate that half of the variation (50.08%) is explained by niche divergence, in agreement with results of the SAMOVA analysis. In addition to the complete lack of shared haplotypes, the high *F*_ST_ values for the pairwise comparisons between populations from the east v. west of the TMVB indicate that there is no gene flow between these two regions. Furthermore, the SAMOVA and test of niche divergence indicate that the two clades could be diverged via ecological speciation as they clearly fulfil Svensson's [1] criteria of ecological speciation. This observed pattern could be the result of historical divergence, where the genetic drift may have acted in the smaller western populations, allowing divergent selection to act more rapidly given the high degree of reproductive isolation.

Climatic spatial resistance surfaces for the present and the Last Glacial Maximum showed some corridors between neighbouring populations in the eastern region of the TMVB, but no connectivity was maintained with the populations in the western region of the TMVB (Fig. 4A, B, C). The spatial genetic correlations showed a positive and significant (present: *r* = 0.27; *P* = 0.005; CCSM: *r* = 0.52; *P* = 0.001 MIROC: *r* = 0.28; *P* = 0.004) correlation between genetic *F*_ST_/(1-*F*_ST_) pairwise distances and spatial resistance distances among populations, both present-day and in the LGM, indicating that the populations have been isolated since the LGM (Ruiz-Sanchez & Specht, 2013[22]) with no gene flow between them, except for immediately adjacent populations (Fig. 4).

These results contradict those reported by Wiens [9] and Velo-Antón et al. [17], where the LGM permitted a continuous distribution that could increase the gene flow among populations. Instead, our results are more similar to those reported by Bryson & Riddle [27], where tropical montane specialists persisted in isolation over longer periods of time, corroborated by empirical studies of vegetation change in central and western Mexico over the last 86,000 years [51], [52]. Our previous study [22] revealed relatively old divergence times between the eastern and western clades (see their Fig. 3) indicating that the uplift of the TMVB at different times in the past may have influenced the genetic and ecological diversification of *N. parviflora*. The Neogene formation of the TMVB with different episodes of volcanism [23], [24] appears to have promoted diversification via habitat fragmentation and the resulting isolation of populations for different plant and animal lineages [20], [25]–[27], [53], [54].

Our results provide evidence for differentiation among populations reflecting habitat preference among extant populations, and some of these segregated populations could be designated as separately evolving lineages given that gene flow appears to be completely lacking [7]. Their occupation of different habitats could further limit dispersal between distant populations [49], even more than would be expected as a result of distance alone. Ecological divergence could represent an important stage in the process of lineage separation and speciation [1], [55]. Ecological speciation could be the key process in the speciation of *N. parviflora* along the Trans-Mexican Volcanic Belt, a conclusion supported by our ecological analyses and the observed morphological differences identified between the eastern v. western lineages. A complete taxonomic description of the new *Nolina* species is currently in preparation (Ruiz-Sanchez et al. in prep.).
